# Post-treatment control or treated controllers? Viral remission in treated and untreated primary HIV infection

**DOI:** 10.1097/QAD.0000000000001382

**Published:** 2017-02-01

**Authors:** Genevieve E. Martin, Morgane Gossez, James P. Williams, Wolfgang Stöhr, Jodi Meyerowitz, Ellen M. Leitman, Philip Goulder, Kholoud Porter, Sarah Fidler, John Frater

**Affiliations:** a Nuffield Department of Medicine, Peter Medawar Building for Pathogen Research, University of Oxford, Oxford; b MRC Clinical Trials Unit at University College London, London; c Department of Paediatrics, University of Oxford, Oxford, UK; d Harvard Medical School, Boston, Massachusetts, USA; e HIV Pathogenesis Programme, Doris Duke Medical Research Institute, University of KwaZulu-Natal, Durban, South Africa; f Research Department of Infection and Population Health, University College London; g Division of Medicine, Wright Fleming Institute, Imperial College, London; h The Oxford Martin School; i Oxford National Institute of Health Research Biomedical Research Centre, Oxford, UK.

**Keywords:** antiretroviral therapy, ELISpot, HIV, HIV DNA, natural history, post-treatment control, T lymphocytes

## Abstract

**Objective(s):**

An HIV cure will impose aviraemia that is sustained following the withdrawal of antiretroviral therapy (ART). Understanding the efficacy of novel interventions aimed at curing HIV requires characterization of both natural viral control and the effect of ART on viral control after treatment interruption.

**Design:**

Analysis of transient viral control in recent seroconverters in the Short Pulse AntiRetroviral Therapy at Acute Seroconversion trial.

**Methods:**

We compared untreated and treated HIV seroconverters (*n* = 292) and identified periods of control (plasma HIV RNA < 400 copies/ml for ≥16 weeks off therapy) in 7.9% of ART-naive participants, and in 12.0% overall. HIV DNA was measured by qPCR, and HIV-specific CD8^+^ responses were measured by enzyme-linked immunosorbent spot assay (ELISpot). T-cell activation and exhaustion were measured by flow cytometry.

**Results:**

At baseline, future controllers had lower HIV DNA, lower plasma HIV RNA, higher CD4^+^ : CD8^+^ ratios (all *P* < 0.001) and higher CD4^+^ cell counts (*P* < 0.05) than noncontrollers. Among controllers, the only difference between the untreated and those who received ART was higher baseline HIV RNA in the latter (*P* = 0.003), supporting an added ART effect.

**Conclusion:**

Consideration of spontaneous remission in untreated individuals will be critical to avoid overestimating the effect size of new interventions used in HIV cure studies.

## Abstract 2

Supplemental Digital Content is available in the text

## Introduction

Strategies to achieve an HIV cure by implementing interventions to deplete the HIV reservoir – a pool of latently infected cells containing transcriptionally repressed viral DNA – are entering clinical trials. In the absence of a validated biomarker to prove cure, a key outcome measure in these studies is the time to detection of viral RNA, or ‘viral rebound’, in plasma after stopping antiretroviral therapy (ART) in an analytic treatment interruption [1–5].

Patients with primary HIV infection (PHI) are of particular interest, as with lower reservoir sizes [6–9] and preserved immunity [6,10–13] with less immune escape [14–16], there may be increased prospect of achieving remission. ART alone, when initiated during PHI, may induce remission, as described for post-treatment controllers (PTCs) in the VISCONTI cohort [17]. However, to understand the impact of ART – or any other intervention – on remission requires characterization of the time from seroconversion to a detectable HIV plasma viral load in untreated patients. Treatment of PHI is now recommended in revised international clinical guidelines [18,19] meaning that randomized trials with a ‘no treatment’ arm cannot be undertaken. As such, we must turn to high-quality historical data to assist interpretation of treatment interruption studies.

The ‘Short Pulse AntiRetroviral Therapy at Acute Seroconversion’ (SPARTAC) trial [20,21] was a randomized controlled trial of 0, 12 or 48 weeks of ART initiated during PHI. The study design provided the unusual opportunity to compare viral load dynamics in treated and untreated seroconverters. We turned to SPARTAC to understand whether reports of PTC and virological remission following treatment interruption might be conflated by natural transient delays to detectable viraemia in untreated seroconverters.

## Methods

### Participants and trial design

SPARTAC (EudraCT number: 2004-000446-20) was a multicentre randomized controlled trial of short-course ART (12 or 48 weeks ART, vs no immediate ART) initiated during PHI [20]. For all analyses, baseline refers to the date of randomization. Of 366 participants, 74 were excluded for logistical reasons, leaving 292 participants who were included in this analysis. Participants were assessed according to whether they received 12 or less vs more than 12 weeks ART, compared with no immediate ART. This same 12 week cut-off was used in a previous ‘as received’ analysis of SPARTAC [22].

Within this ‘at risk’ group, we identified controllers who experienced a period of remission which was defined for all as HIV RNA less than 400 copies/ml, on two measurements at least 16 weeks apart, with the period of remission starting within 1 year of randomization or ART cessation (untreated and treated groups, respectively). We included participants who experienced a viral blip of any magnitude at a single time point. Time of viral rebound was the first of at least two consecutive measurements more than 400 copies/ml.

### HIV DNA quantification

Total HIV DNA was quantified at baseline (where there were sufficient samples available; *n* = 200) by a previously described qPCR assay [21,23].

### CD8^+^ T-cell ELISpot assays

HIV Gag-specific CD8^+^ T-cell responses were measured by IFN-γ ELISpot assays to overlapping peptides using methods described elsewhere [24,25].

### Flow cytometry

The expression of exhaustion (PD-1, Tim-3 and Lag-3) and activation [CD25, CD38, CD69 and human leucocyte antigen (HLA) DR] markers on CD4^+^ and CD8^+^ T cells was measured on cryopreserved samples (refer to Supplemental Digital Content 1 for antibodies used). Data were acquired on a MacsQuant Analyser (Miltenyi, Bergisch Gladbach, Germany) and analysed using FlowJo Version 10.0.7 or 10.8.0r1 (Treestar, Ashland, Oregon, USA).

### Statistical analyses

Categorical variables were compared using χ^2^ or Fisher–Freeman–Halton exact test as appropriate. Continuous variables were compared across three groups using the Kruskal–Wallis test or analysis of variance (ANOVA). Pairwise comparisons were made using Mann–Whitney or Student's *t* test. Duration of viral control was assessed using Kaplan–Meier estimates. For all tests, *P* values less than 0.05 were considered statistically significant. Analyses were performed using R version 3.2.2. Plots were drawn using GraphPad Prism (GraphPad Software, La Jolla, California, USA) version 6.0f.

## Results and Discussion

### Transient control of viraemia is evident in untreated primary HIV infection

Most studies of virological control have focused on ‘elite’ and PTCs [17,26,27]. We set out to explore a different question – are there individuals who experience transient viral control during untreated PHI, and how does this compare with post-ART remission?

Our analysis included 292 of 366 participants recruited to the SPARTAC trial who had sufficient HIV RNA sampling and, if treated, were virologically suppressed prior to treatment interruption. Time on ART is analysed ‘as received’ rather than according to randomization arm and stratified as 0, 12 or less or more than 12 weeks of ART. Throughout this analysis, HIV RNA measurements less than 400 copies/ml are considered ‘suppressed’ as this was the lower limit of detection for assays conducted at South African and Ugandan trial sites. As such, this was the lowest HIV RNA threshold that could be applied across all samples.

Considering these participants, regardless of ART use, 35 of 292 (12.0%) experienced a period of suppressed viraemia (HIV RNA < 400 copies/ml) of at least 16 weeks while off therapy and are termed ‘controllers’ for this report. Of the 126 participants who did not receive immediate ART, 10 (7.9%) experienced a period of spontaneous viral control within 1 year of randomization. Among individuals who received short-course ART (*n* = 80 ≤ 12 weeks; *n* = 86 > 12 weeks) and underwent treatment interruption, 25 (15.1%) experienced viral remission in the subsequent year.

PTCs have been almost exclusively identified among individuals who initiated ART during PHI, suggesting an impact of early ART on long-term viral control [17]. We found some evidence of greater frequency of control in individuals who had received more than 12 weeks ART, when compared across all three groups, although this was not statistically significant (*P* = 0.06; Table 1). In the best characterized cohort of PTCs to date (VISCONTI), patients received a median of 3 years ART prior to treatment interruption [17]. Twelve weeks of ART is likely to be too short to induce durable PTC, and it is possible that the participants who received 12 weeks or less ART in this analysis were more similar to untreated controllers than those who received more than 12 weeks ART (as also indicated by the primary outcome analysis of SPARTAC, which was based on clinical progression [20]). Accordingly, we performed all comparisons across the three groups rather than combining the two ART arms. Of note, if the 12 week or less group was excluded from the analysis of control, there were significantly more controllers following ART compared with no treatment (18.6% vs 7.9%; *P* = 0.03). In our previous analysis of viral rebound in only the treated participants in SPARTAC, longer treatment duration was significantly associated with slower viral rebound within 12 weeks of stopping ART consistent with these findings [22].

**Table 1:** Characteristics of controllers identified in Short Pulse AntiRetroviral Therapy at Acute Seroconversion.

	Total, *n* = 292	≤12 weeks ART, *n* = 80	>12 weeks ART, *n* = 86	No ART, *n* = 126	*P* value
No. of controllers (% total participants)	35 (12.0)	9 (11.3)	16 (18.6)	10 (7.9)	0.06
Duration of short course ART [weeks; median (IQR)]	–	11.9 (11.8–12.0)	47.8 (47.3–47.9)	–	–
Interval between estimated serconversion and randomization [days; median (IQR)]	–	92.0 (64.0–106)	97.5 (58.0–125)	95.5 (76.8–116)	0.64
Viral subtype					0.16
B	13	2	6	5	
C	18	5	10	3	
Other (A1, B/C, B/F and D)	4	2	0	2	
Country^a^					0.38
South Africa/Uganda	20	6	10	4	
United Kingdom	11	3	3	5	
Other (Australia/Brazil)	4	0	3	1	
Sex					1
Male	13	3	6	4	
Female	22	6	10	6	
Age [years; median (IQR)]	–	40 (25–47.5)	27.5 (21.3–46.5)	32 (26–35.5)	0.33
HLA alleles^b^					
≥1 protective class I allele	11	1	7	3	0.54
≥1 disease-susceptible class I allele	11	5	5	1	0.08
B^*^35 : 01 allele	2	0	2	0	–
Duration of remission (weeks)					–
<26	1	0	0	1	
26–52	10	6	3	1	
52–104	8	0	7	1	
>104	16	3	6	7	
No. without viral rebound at end of follow-up	8 (2.7)	1 (1.3)	5 (5.8)	2 (1.6)	–

Statistical tests were performed across all three groups. Categorical variables, including number of controllers, were compared using χ^2^ test or Fisher–Freeman–Halton exact test as appropriate; continuous variables were compared using Kruskal–Wallis test. IQR, interquartile range. Duration of remission was calculated between date of randomization (untreated arm) or ART cessation (treated arms) and viral rebound. ART, antiretroviral therapy. Protective class I alleles were defined as B^*^27 : 05, B^*^57 : 01 for subtype B and B^*^57 : 02, B^*^57 : 03, B^*^81 : 01, B^*^58 : 01 for subtype C. Disease-susceptible class I alleles were defined as B^*^35 : 01, B^*^07 : 02 for subtype B and B^*^58 : 02, B^*^18 : 01 for subtype C [24,28,29].

^a^No controllers were identified from Ireland, Spain or Italy.

^b^Analysis performed for participants with subtype B or C virus only.

A strength of this analysis is the ability to quantify viral remission among treated and untreated individuals with PHI in a well characterized and frequently sampled randomized study. Several studies of ART initiated during PHI have demonstrated the presence of individuals who transiently control viral replication post-treatment interruption [30–35]. None of these previous studies included an untreated arm, thus limiting their ability to evaluate the added impact of ART on the presence of transient viral control. An analysis of the Quest study (in which participants underwent treatment interruption following at least 72 weeks ART initiated during PHI) [32] used data from untreated individuals from a separate PHI cohort (CASCADE) as controls. That analysis showed no significant difference in the frequency of transient viral control between the two studies [36], although a different HIV RNA cut-off (1000 copies/ml) and duration of analysis were used.

### Duration of remission in treated and untreated controllers

We next looked to see whether the duration of viral control varied between controllers who did or did not receive ART. There were more participants with over 1 year of remission among those receiving more than 12 weeks ART compared with 12 weeks or less or no ART [13 (15.1% of all participants) vs 3 (3.8%) vs 8 (6.3%), respectively (Table 1)]. Eight controllers (five of whom had received >12 weeks ART) experienced undetectable HIV RNA until the end of follow-up [median 192 weeks (interquartile range 165–202)]. There was, however, no statistically significant difference between the duration of remission between the three groups when including the full duration of follow-up (*P* = 0.22; log rank). Interestingly, when just considering the controllers, untreated participants were more likely to experience sustained control more than 104 weeks (7/10) compared with the treated controllers for whom rebound was more evenly distributed over the assessment period, and which may have implications for the underlying mechanisms.

### Controllers have more favourable baseline clinical characteristics than noncontrollers

**Fig. 1:**
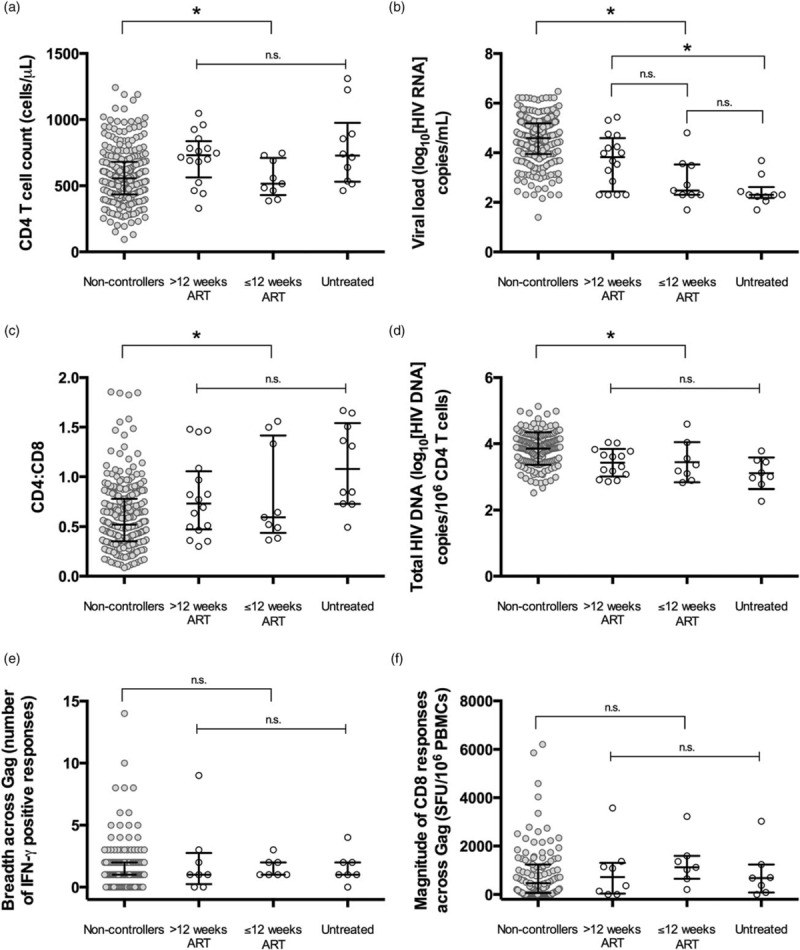
Clinical characteristics of controllers identified in Short Pulse AntiRetroviral Therapy at Acute Seroconversion.

In comparison with the noncontrollers (regardless of treatment group), future controllers had more favourable baseline clinical characteristics with higher CD4^+^ T-cell counts (median 700 vs 557 cells/μl), lower plasma HIV RNA (median 2.70 vs 4.59 log_10_ copies/ml), higher CD4^+^-to-CD8^+^ ratio (median 0.77 vs 0.52) and lower total HIV DNA [mean 3.35 vs 3.85 log_10_ copies/10^6^ CD4^+^ T cells (Fig. 1a–d; Table, Supplemental Digital Content 2 contains values)]. These findings are consistent with previous studies of viral control during PHI with [30,33,34,37] and without [38,39] treatment.

Baseline clinical variables of controllers (three groups shown with open circles) and noncontrollers (green, shaded circles) identified in Short Pulse AntiRetroviral Therapy at Acute Seroconversion. Bars shown indicate the mean (SD) for total HIV DNA (d) and median (interquartile range) for all other parameters (a–c, e, f). CD4^+^ cell count (a), viral load (b), CD4^+^ to CD8^+^ ratio [(C) all total *n* = 292], as well as breadth (e) and magnitude [(F) both total *n* = 145] of CD8^+^ responses across Gag were compared between noncontrollers and combined controller groups using the Mann–Whitney test. Total HIV DNA [(d) total *n* = 200] was compared between these two groups using Student's *t* test. Comparisons between three controller groups were made using Kruskal–Wallis (a–c, e, f) and ANOVA (d) tests. For viral load, this was significantly different (*P* = 0.006), and pairwise comparisons shown (b) were made between groups using the Mann–Whitney test. ^∗^*P* < 0.05.

### Spontaneous controllers have lower baseline HIV RNA than those who received more than 12 weeks antiretroviral therapy

When focusing on just the controllers, untreated participants had similar baseline CD4^+^ cell counts, CD4^+^ : CD8^+^ ratios and HIV DNA levels to those who controlled following treatment interruption, but had significantly lower baseline HIV RNA than those who had received more than 12 weeks ART [median 2.30 vs 3.82 log_10_ copies/ml, respectively (*P* = 0.002; Fig. 1b; Table, Supplemental Digital Content 2)]. The interval between seroconversion and baseline was similar between treatment groups and did not explain this finding (Table 1). This difference in baseline HIV RNA supports an additional impact of ART in inducing viral remission in some individuals who otherwise may not control viral replication, providing evidence for post-treatment control as a distinct phenomenon.

### Similar demographics and immunological characteristics in antiretroviral therapy–receiving and spontaneous controllers.

Next, we compared the demographic and immunological characteristics of untreated and treated controllers (*n* = 35). We found no evidence for demographic differences between untreated and treated controllers in terms of sex, viral subtype, country of origin and age (Table 1). Because CD8^+^ T-cell responses are associated with clinical progression [40] and drive durable spontaneous (or ‘elite’) control, we looked for the presence of protective HLA Class I alleles amongst controllers identified in this study. The proportion of controllers with protective or disease-susceptible HLA Class I alleles was similar between treatment groups. Two controllers carried HLA B^∗^35 alleles, which has been observed amongst PTCs in the VISCONTI case series [17]. As a measure of CD8^+^ recognition of HIV, we measured CD8^+^ T-cell responses to HIV peptides by Gamma Interferon ELISpot. We assessed responses across HIV Gag, which did not differ between treated and untreated controllers and were similar in breadth (Fig. 1e) and magnitude (Fig. 1f) to those measured in noncontrollers. The percentage of CD4^+^ and CD8^+^ T cells expressing markers of exhaustion (PD-1, Tim-3 and Lag-3) and activation (HLA-DR, CD69, CD25 and CD38) at baseline also did not differ between treatment groups (data not shown).

## Conclusion

There are two key findings to this analysis. One is the demonstration of long periods of transient viral remission in a substantial proportion of untreated individuals after PHI, which may conflate data from those who transiently control after ART interruption. The second finding confirms previous reports of beneficial baseline characteristics associated with future virological control and supports an additional impact of ART during PHI.

Following the results of the START trial, which provided evidence of clear clinical benefit in starting ART irrespective of CD4^+^ cell count [41], untreated controls cannot be included in future HIV trial protocols. The use of uncontrolled treatment interruption studies to measure the success of potentially curative strategies means that modest delays in viral rebound may be attributed to an intervention. Accordingly, consideration of spontaneous remission in PHI will be critical to avoid overestimating the effect size of interventions used in treatment interruption studies.

## Acknowledgements

We thank the participants of SPARTAC. The SPARTAC Trial Investigators: Trial Steering Committee: Independent Members – A. Breckenridge (Chair), P. Clayden, C. Conlon, F. Conradie, J. Kaldor^∗^, F. Maggiolo, F. Ssali, Country Principal Investigators – D.A. Cooper, P. Kaleebu, G. Ramjee, M. Schechter, G. Tambussi, J.M. Miro, J. Weber. Trial Physician: S. Fidler. Trial Statistician: A. Babiker. Data and Safety Monitoring Committee (DSMC): T. Peto (Chair), A. McLaren (in memoriam), V. Beral, G. Chene, J. Hakim. Co-ordinating Trial Centre: Medical Research Council Clinical Trials Unit, London (A. Babiker, K. Porter, M. Thomason, F. Ewings, M. Gabriel, D. Johnson, K. Thompson, A. Cursley^∗^, K. Donegan^∗^, E. Fossey^∗^, P. Kelleher^∗^, K. Lee^∗^, B. Murphy^∗^, D. Nock^∗^). Central Immunology Laboratories and Repositories: The Peter Medawar Building for Pathogen Research, University of Oxford, UK (R. Phillips, J. Frater, L. Ohm Laursen^∗^, N. Robinson, P. Goulder, H. Brown). Central Virology Laboratories and Repositories: Jefferiss Trust Laboratories, Imperial College, London, UK (M. McClure, D. Bonsall^∗^, O. Erlwein^∗^, A. Helander^∗^, S. Kaye, M. Robinson, L. Cook^∗^, G. Adcock^∗^, P. Ahmed^∗^). Clinical Endpoint Review Committee: N. Paton, S. Fidler. Investigators and Staff at Participating Sites: Australia: St Vincents Hospital, Sydney (A. Kelleher), Northside Clinic, Melbourne (R. Moore), East Sydney Doctors, Sydney (R. McFarlane), Prahran Market Clinic, Melbourne (N. Roth), Taylor Square Private Clinic, Sydney (R. Finlayson), The Centre Clinic, Melbourne (B. Kiem Tee), Sexual Health Centre, Melbourne (T. Read), AIDS Medical Unit, Brisbane (M. Kelly), Burwood Rd Practice, Sydney (N. Doong), Holdsworth House Medical Practice, Sydney (M. Bloch), Aids Research Initiative, Sydney (C. Workman). Coordinating Centre in Australia: Kirby Institute University of New South Wales, Sydney (P. Grey, D.A. Cooper, A. Kelleher, M. Law). Brazil: Projeto Praca Onze, Hospital Escola Sao Francisco de Assis, Universidade federal do Rio de Janeiro, Rio de Janeiro (M. Schechter, P. Gama, M. Mercon^∗^, M. Barbosa de Souza, C. Beppu Yoshida, J.R. Grangeiro da Silva, A. Sampaio Amaral, D. Fernandes de Aguiar, M. de Fatima Melo, R. Quaresma Garrido). Italy: Ospedale San Raffaele, Milan (G. Tambussi, S. Nozza, M. Pogliaghi, S. Chiappetta, L. Della Torre, E. Gasparotto), Ospedale Lazzaro Spallanzani, Roma (G. DOffizi, C. Vlassi, A. Corpolongo). South Africa: Cape Town: Desmond Tutu HIV-1 Centre, Institute of Infectious Diseases, Cape Town (R. Wood, J. Pitt, C. Orrell, F. Cilliers, R. Croxford, K. Middelkoop, L.G. Bekker, C. Heiberg, J. Aploon, N. Killa, E. Fielder, T. Buhler). Johannesburg: The Wits Reproductive Health and HIV-1 Institute, University of Witswatersrand, Hillbrow Health Precinct, Johannesburg (H. Rees, F. Venter, T. Palanee), Contract Laboratory Services, Johannesburg Hospital, Johannesburg (W. Stevens, C. Ingram, M. Majam, M. Papathanasopoulos). Kwazulu-Natal: HIV-1 Prevention Unit, Medical Research Council, Durban (G. Ramjee, S. Gappoo, J. Moodley, A. Premrajh, L. Zako). Uganda: Medical Research Council/Uganda Virus Research Institute, Entebbe (H. Grosskurth, A. Kamali, P. Kaleebu, U. Bahemuka, J. Mugisha^∗^, H.F. Njaj^∗^). Spain: Hospital Clinic-IDIBAPS, University of Barcelona, Barcelona (J.M. Miro, M. Lopez-Dieguez^∗^, C. Manzardo, J. Ambrosioni, D. Nicolas, J.A. Arnaiz, T. Pumarola, M. Plana, M. Tuset, M.C. Ligero, M.T. Garca, T. Gallart, J.M. Gatell). UK and Ireland: Royal Sussex County Hospital, Brighton (M. Fisher, K. Hobbs, N. Perry, D. Pao, D. Maitland, L. Heald), St James's Hospital, Dublin (F. Mulcahy, G. Courtney, S. O’Dea, D. Reidy), Regional Infectious Diseases Unit, Western General Hospital and Genitourinary Dept, Royal Infirmary of Edinburgh, Edinburgh (C. Leen, G. Scott, L. Ellis, S. Morris, P. Simmonds), Chelsea and Westminster Hospital, London (B. Gazzard, D. Hawkins, C. Higgs), Homerton Hospital, London (J. Anderson, S. Mguni), Mortimer Market Centre, London (I. Williams, N. De Esteban, P. Pellegrino, A. Arenas-Pinto, D. Cornforth^∗^, J. Turner^∗^), North Middlesex Hospital (J. Ainsworth, A. Waters), Royal Free Hospital, London (M. Johnson, S. Kinloch, A. Carroll, P. Byrne, Z. Cuthbertson), Barts & the London NHS Trust, London (C. Orkin, J. Hand, C. De Souza), St Marys Hospital, London (J. Weber, S. Fidler, E. Hamlyn, E. Thomson^∗^, J. Fox^∗^, K. Legg, S. Mullaney^∗^, A. Winston, S. Wilson, P. Ambrose), Birmingham Heartlands Hospital, Birmingham (S. Taylor, G. Gilleran). Imperial College Trial Secretariat: S. Keeling, A. Becker. Imperial College DSMC Secretariat: C. Boocock. (^∗^ Left the study team before the trial ended.).

The SPARTAC Trial was funded by The Wellcome Trust (grant no. 069598/Z/02/Z), J.F. is funded by the Medical Research Council, G.M. is funded by the Nuffield Department of Medicine, the Clarendon Fund and the General Sir John Monash Foundation.

Author contributions: Conceived and designed the experiments – G.M., W.S., K.P., S.F., J.F. Performed the experiments – G.M., M.G., J.W., J.M., E.L. Analysed the data – G.M., W.S., K.P., J.F. Contributed reagents/materials/analysis tools: P.G. Wrote the article: G.M., W.S., K.P., S.F., J.F.

### Conflicts of interest

There are no conflicts of interest.

## Supplementary Material

Supplemental Digital Content
