# Supplementary material for: Post-treatment control or treated controllers? Viral remission in treated and untreated primary HIV infection
Source: AIDS. 2017 Feb 1;31(4):477–84. doi: 10.1097/QAD.0000000000001382 (PMC5278888; doi:10.1097/QAD.0000000000001382)
Supplement: Supplemental Digital Content [file aids-31-477-s001.docx]

**Supplemental Digital Content 1.**

**Antibodies used for the measurement of CD4 and CD8 T cell activation and exhaustion**

CD4 VioGreen (VIT4), CD8 APC (BW135/80), CD25 PE (3G10), CD38 PE-Vio770 (IB6), CD69 FITC (FN50), HLA-DR PerCP (AC122; all Miltenyi, Begisch Gladbach, Germany), Tim-3 PE (344823; R&D systems, Minneapolis, MN), Lag-3 PerCPeFluor710 (3D5223H) and PD-1 PE-Cy7 (eBioJ105) and CD3 eFluor450 (UCHTI; all eBiosciences, San Diego, CA) or VioBlue (BW264/56, Miltenyi). Dead cells were excluded using a viability dye (Near-IR; Life Technologies, Calsbad, CA).

**Supplemental Digital Content 2.**

**Table: Baseline immunological and virological characteristics of non-controllers and controllers according to ART duration.**

|  | **Non-controllers** | **Controllers** | | | |
| --- | --- | --- | --- | --- | --- |
|  |  | **All treatment groups (combined)** | **>12 weeks ART** | **≤12 weeks ART** | **Untreated** |
| CD4 T cell count (cells/μL) | 557 [434 – 681] | 700 [515 – 738] | 731 [564 – 837] | 515 [429 – 710] | 728 [531 – 976] |
| Viral load (log_10_[HIV RNA] copies/mL) | 4.59 [3.95 – 5.19] | 2.70 [2.30 – 3.98] | 3.82 [2.44 – 4.59] | 2.47 [2.30 – 3.53] | 2.30 [2.18 – 2.62] |
| CD4:CD8 ratio | 0.52 [0.35 – 0.78] | 0.77 [0.50 – 1.36] | 0.73 [0.47 – 1.06] | 0.60 [0.47 – 1.42] | 1.08 [0.73 – 1.54] |
| Total HIV DNA (log_10_[HIV DNA] copies/10^6^ CD4 T cells) | 3.85 (0.49) | 3.35 (0.49) | 3.43 (0.42) | 3.44 (0.61) | 3.11 (0.47) |
| Breadth of ELISpot response across Gag (number of IFN-ϒ positive responses) | 1.0 [1.0 – 2.0] | 1.0 [1.0 – 2.0] | 1.0 [0.3 – 2.8] | 1.0 [1.0 – 2.0] | 1.0 [1.0 – 2.0] |
| Magnitude of ELISpot response across Gag (SFU/10^6^ PBMCs) | 466 [60.0 – 1240] | 862 [190 – 1361] | 722 [35.0 – 1310] | 1120 [654 – 1593] | 676 [86.0 – 1240] |

**Table: Baseline immunological and virological characteristics of non-controllers and controllers according to ART duration..**

Values for Total HIV DNA are shown as mean (standard deviation). For all other parameters the values shown are medians [interquartile range].
